# Enhancer of Zeste Homolog 2 (EZH2) Mediates Glucolipotoxicity-Induced Apoptosis in β-Cells

**DOI:** 10.3390/ijms21218016

**Published:** 2020-10-29

**Authors:** Tina Dahlby, Christian Simon, Marie Balslev Backe, Mattias Salling Dahllöf, Edward Holson, Bridget K. Wagner, Marianne Böni-Schnetzler, Michal Tomasz Marzec, Morten Lundh, Thomas Mandrup-Poulsen

**Affiliations:** 1 Department of Biomedical Sciences, University of Copenhagen, DK-2200 Copenhagen, Denmark; tinad@sund.ku.dk (T.D.); mariebacke8@gmail.com (M.B.B.); mattiassd@gmail.com (M.S.D.); michal@sund.ku.dk (M.T.M.); lundh@sund.ku.dk (M.L.); 2 Novo Nordisk Foundation Center for Protein Research, University of Copenhagen, DK-2200 Copenhagen, Denmark; christian.simon@cpr.ku.dk; 3 Broad Institute of MIT and Harvard, Cambridge, MA 02142, USA; eholson@kdactherapeutics.com (E.H.); bwagner@broadinstitute.org (B.K.W.); 4 Department of Biomedicine, University Hospital and University of Basel, 4031 Basel, Switzerland; marianne.boeni@unibas.ch

**Keywords:** GLT, histone deacetylases, histone methyltransferase, diabetes, insulin secretion, ER stress, NFκB

## Abstract

Selective inhibition of histone deacetylase 3 (HDAC3) prevents glucolipotoxicity-induced β-cell dysfunction and apoptosis by alleviation of proapoptotic endoplasmic reticulum (ER) stress-signaling, but the precise molecular mechanisms of alleviation are unexplored. By unbiased microarray analysis of the β-cell gene expression profile of insulin-producing cells exposed to glucolipotoxicity in the presence or absence of a selective HDAC3 inhibitor, we identified Enhancer of zeste homolog 2 (EZH2) as the sole target candidate. β-Cells were protected against glucolipotoxicity-induced ER stress and apoptosis by EZH2 attenuation. Small molecule inhibitors of EZH2 histone methyltransferase activity rescued human islets from glucolipotoxicity-induced apoptosis. Moreover, EZH2 knockdown cells were protected against glucolipotoxicity-induced downregulation of the protective non-canonical Nuclear factor of kappa light polypeptide gene enhancer in B-cells (NFκB) pathway. We conclude that EZH2 deficiency protects from glucolipotoxicity-induced ER stress, apoptosis and downregulation of the non-canonical NFκB pathway, but not from insulin secretory dysfunction. The mechanism likely involves transcriptional regulation via EZH2 functioning as a methyltransferase and/or as a methylation-dependent transcription factor.

## 1. Introduction

Type 2 diabetes (T2D) arises when pancreatic β-cell insulin secretion fails to meet insulin demands, which increase due to insulin resistance secondary to obesity [1]. The predisposition to develop T2D is polygenetic and conferred by combinations of multiple genetic variants mainly encoding β-cell regulatory proteins. Environmental factors such as overnutrition and inadequate physical activity significantly contribute to T2D etiology by shifting the energy-balance towards caloric storage and obesity, and by affecting gene transcription by epigenetic mechanisms [2].

Nutrient overload causes β-cell impairment via toxic effects of elevated blood saturated non-esterified fatty acids (NEFAs) and glucose, termed glucolipotoxicity (GLT). NEFAs induce apoptosis [3,4] and inhibit glucose-stimulated insulin-secretion (GSIS) [4,5] in β-cell lines and rat and human islets in vitro. Elevated concentrations of NEFAs infused in humans cause impaired β-cell function [6,7]. Likewise, elevated concentrations of glucose reduce β-cell function in vivo [8], and reduce GSIS, insulin content and insulin gene expression and induce apoptosis in vitro [3,9,10], and exposure of β-cell lines to the NEFA palmitate alters gene expression and histone marks in parallel to impairing insulin secretion [11].

Histone deacetylases (HDACs) catalyze the removal of acetyl groups from lysine residues in histone tails and in >4500 cytosolic proteins, including transcription factors [12]. In the pancreatic β-cell, HDACs 1 and 3 in particular mediate functional impairment and apoptosis in response to inflammatory and glucolipotoxic stress [13,14,15,16,17,18,19,20]. Pan-HDAC and HDAC1- and 3-selective small-molecule HDAC-inhibitors (HDACi) prevent inflammation-induced β-cell dysfunction and apoptosis in cell- and animal-models of diabetes [14,18,19,20] by hyperacetylation, and thereby inactivation, of the master proinflammatory transcription factor Nuclear factor of kappa light polypeptide gene enhancer in B-cells (NFκB) p65 subunit [20].

Additionally, HDACi prevent GLT-induced β-cell apoptosis in vitro and in vivo [15,16,17]. In GLT-exposed β-cells, selective histone deacetylase 3 inhibition (HDAC3i) alleviates proapoptotic endoplasmic reticulum (ER) stress as evidenced by reduced C/EBP homologous protein (Chop) and c-Jun N-terminal kinase (JNK) activation [16], but the precise molecular mechanisms are unknown. Thus, the aim of the current study was to identify such novel targets and mechanisms by which β-cell dysfunction and apoptosis are reverted by HDAC3i by employing in silico and in vitro models. We identified the histone-lysine *N*-methyltransferase (HMT) Enhancer of zeste homolog 2 (EZH2) as the sole target candidate protein associated with genes regulated by both GLT and HDAC3i in the β-cell line INS-1E.

EZH2 displays multifaceted functions in cells. The canonical role of EZH2 is co-factor dependent transcriptional repression through three sequential methylations on lysine 27 of the histone-3 tail (H3K27me3) (reviewed in [21]). The core co-factors Suppressor of Zeste 12 (SUZ12), Embryonic Ectoderm Development (EED) and Histone-Binding Protein Histone-Binding Protein Retinoblastoma-Binding Protein 4 (RbAp48) assemble with EZH2 into the Polycomb Repressive Complex 2 (PRC2). The non-canonical roles of EZH2 include protein regulation through PRC2-subunit independent protein-methylation [22,23], and PRC2-independent and methylation-dependent [24,25] or -independent [26] EZH2 transcriptional activation. Intriguingly, EZH2 is a co-activator of the HDAC-regulated transcription factor NFκB [26]. Canonical NFκB activation has been shown to shift the ER stress response from a protective to a proapoptotic mode [27,28], whereas the *Chop* promoter contains binding sites for the non-canonical NFκB-subunit p52, although it is unknown whether these sites are inhibitory or activating in β-cells [29].

The current available research on the role of EZH2 in the β-cell describes EZH2 as a critical regulator of H3K27me3 marks during pancreatic endocrine specification [30], control of β-cell dedifferentiation [31,32] and as a regulator of β-cell proliferation [32,33]. While homozygous, β-cell specific EZH2 deficiency in *RIP-Cre;Ezh2^f/f^* or *Pdx1-Cre;Ezh2^f/f^* mice induces mild glucose-intolerance and reduced β-cell mass [30,33]; heterozygous *Pdx1-Cre;Ezh2^f/+^* mice display increased β-cell mass [30]. In addition, β cells of pancreatic sections from human T2D donors exhibit reduced H3K27me3 marks [31]. Interpretation of these findings is difficult, since the outcomes in absence of inducible deletion may be confounded by actions on β-cell ontogeny, and the changes observed in the human T2D donors can be construed as both pathogenic and defensive.

We here demonstrate a direct protective role of EZH2 deficiency in insulin producing INS-1E cells and in human islets. First, we demonstrate that knockdown or inhibition of EZH2 protects INS-1E cells and human islets against metabolic (GLT-induced) ER stress and apoptosis, without affecting GLT-induced insulin-secretory dysfunction. We provide mechanistic evidence that EZH2 deficiency protects against GLT-induced downregulation of the non-canonical NFκB pathway.

## 2. Results

### 2.1. EZH2 Is a Transcriptional Node Controlling Genes Regulated by GLT and HDAC3 Inhibition

**Figure 1:**
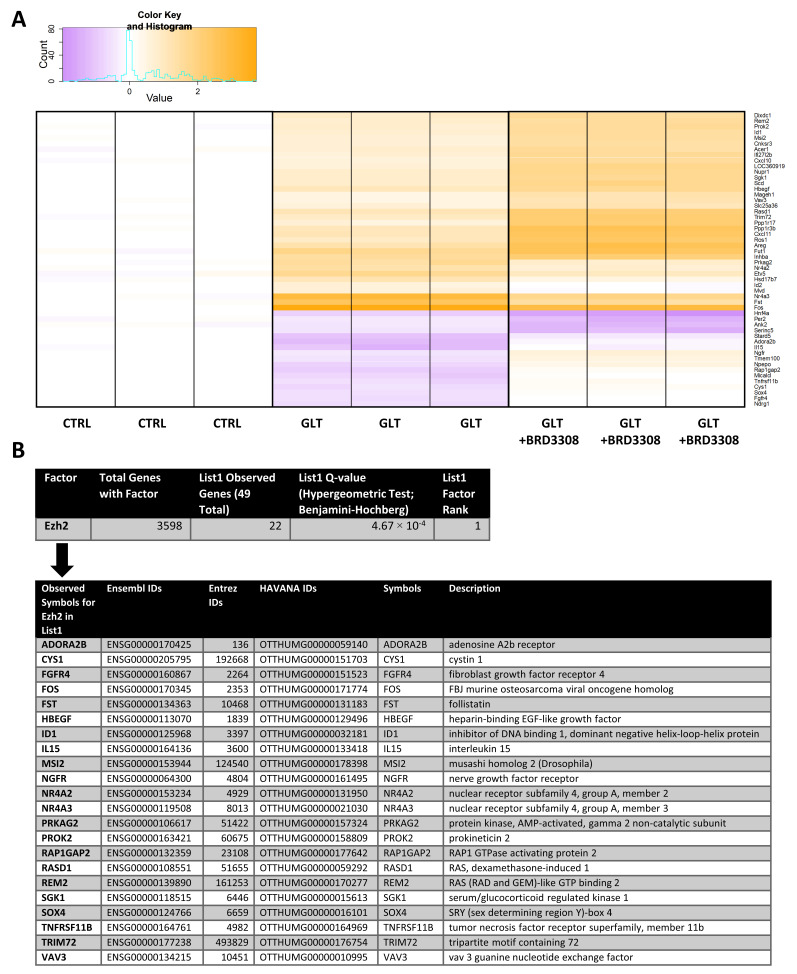
Enhancer of zeste homolog 2 (EZH2) putatively binds gene targets regulated by glucolipotoxicity (GLT) and BRD3308. (**A**) mRNA isolated from INS-1E cells exposed to 25 mmol/L glucose and 0.5 mmol/L palmitate (GLT) with or without 10 μmol/L histone deacetylase 3 (HDAC3) inhibitor BRD3308 was spotted in triplicate onto an Affymetrix gene array chip (*n* = 1 array). On the heatmap, yellow represent upregulated genes and purple represents downregulated genes relative to the control, with the scale shown in the upper left corner. (**A**) shows the expression pattern of the 52 genes identified as regulated both by GLT and by BRD3308. See gene names and regulation patterns in Table S1. (**B**) Genes from (**A**) were analyzed by the ENCODE ChIP-Seq Significance Tool, and only EZH2 was identified as a factor binding 22 of the genes (top panel). The bottom panel shows a description of the subset of genes from (**A**) that was identified to contain EZH2 binding sites. The Tool employs a hypergeometric test with multiple hypothesis correcting using Benjamini–Hochberg to calculate binding significance. List1: Input list of the 52 genes from (**A**).

To investigate the protective mechanisms of HDAC3i in vitro, insulin producing INS-1E cells were exposed to GLT in the presence or absence of the selective HDAC3i BRD3308 for 6 h. We then performed an explorative mRNA microarray to analyze GLT-regulated mRNAs that were affected by the selective HDAC3i. Microarray data were analyzed by an unbiased bioinformatics analysis, avoiding functional enrichment/pathway analyses, to identify relevant gene sets. Fifty-two genes were differentially regulated by both GLT alone and GLT in combination with BRD3308 (Figure 1A and Table S1). In order to link microarray data to relevant transcription factors targeting the 52 differentially regulated genes in our dataset, we used these genes as input for the in silico ENCODE ChIP-Seq Significance Tool [34]. The Tool only produced one single output: Enhancer of zeste homolog 2 (EZH2). EZH2 is a transcription factor and was associated with 22 of 49 genes in our list (Figure 1B), suggesting that EZH2 regulates these genes Three rat-specific genes, *Aminopeptidase O* (*Npepo*), *Interferon, alpha-inducible protein 27 like 2B* (*Ifi27l2b*) and *Similar to alpha-fetoprotein* (*LOC360919*) were lost in the analysis, since the Tool employs a human database. However, substituting the rat-specific genes with their human counterparts, *Aopep*, *Ifi27l2* and *Afp*, respectively, did not change the analysis. The microarray was validated by qPCR of selected genes (Figure S1A–F), and we confirmed that GLT and BRD3308 regulate apoptosis in our cell model (Figure S2).

### 2.2. EZH2 Attenuation Protects Against GLT-Induced Apoptosis

**Figure 2:**
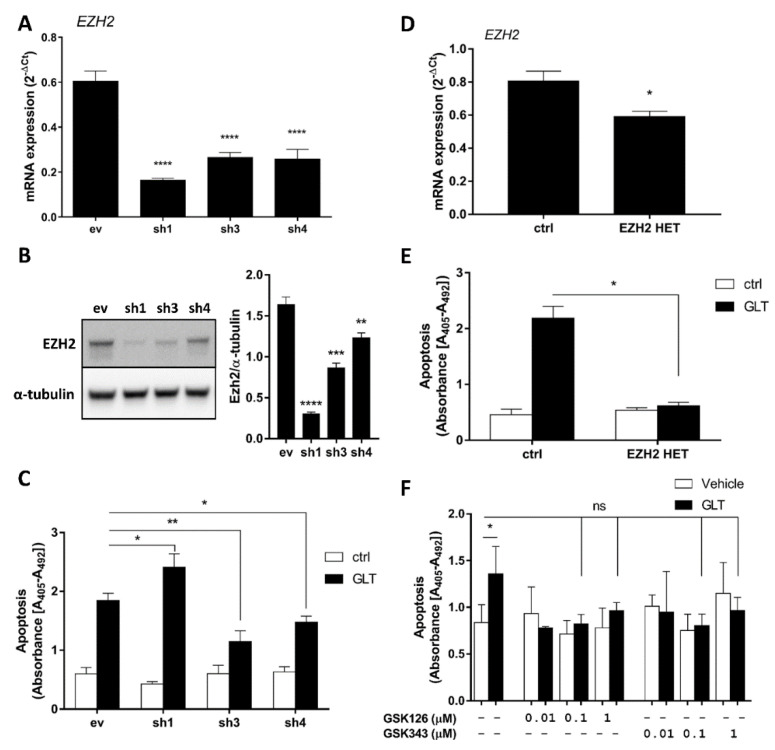
Moderate Enhancer of zeste homolog 2 (EZH2) attenuation protects against glucolipotoxicity (GLT)-induced apoptosis. (**A**) mRNA expression of *Ezh2* was measured by real-time quantitative PCR in INS-1E cells expressing different shRNAs targeting EZH2 (EZH2 KDs; sh1, -3 and -4). Data presented as means + SEM of *n* = 4, analyzed by one-way ANOVA with Sidak’s multiple comparisons test (KDs vs. ev). For (**A**–**C**): ev, INS-1E cells transfected with empty vector; sh1, INS-1E cells transfected with shRNA sequence #1; sh3, INS-1E cells transfected with shRNA sequence #3; sh4, INS-1E cells transfected with shRNA sequence #4. (**B**) EZH2 protein expression was detected by Western blot in EZH2 KDs sh1, -3 and -4. Representative blot of *n* = 10. Data presented as means + SEM of *n* = 10, analyzed by one-way ANOVA with Sidak’s multiple comparisons test (KDs vs. ev). (**C**) Fifty-thousand EZH2 KDs sh1, -3 and -4 were exposed to 25 mmol/L glucose and 0.5 mmol/L palmitate (GLT) for 24 h. Apoptosis was detected as cytoplasmic accumulation of mono- and oligonucleosomes. Data presented as means + SEM of *n* = 5–7, analyzed by one-way ANOVA with Sidak’s multiple comparisons test. (**D**) mRNA expression of *Ezh2* was measured by real-time quantitative PCR in INS-1E cells expressing a gRNA targeting exon 3 of EZH2 (EZH2 HET). Data presented as means + SEM of *n* = 3, analyzed by paired Student’s *t*-test. For (**D**,**E**): ctrl, failed INS-1E CRISPR clone; EZH2 HET, CRISPR/Cas9 modified INS-1E with gRNA targeting EZH2 exon 3. (**E**) Fifty-thousand EZH2 HET cells were exposed to 25 mmol/L glucose and 0.5 mmol/L palmitate (GLT) for 24 h. Apoptosis was detected as above. Data presented as means + SEM of *n* = 4, analyzed by one-way ANOVA with Sidak’s multiple comparisons test. (**F**) Twenty-five human islets in duplicate were exposed to 25 mmol/L glucose and 0.5 mmol/L palmitate (GLT) in the presence or absence of 0.01 μmol/L (*n* = 2) and 0.1–1 μmol/L (*n* = 5) EZH2 inhibitor GSK126 or GSK343 for 72 h. Apoptosis measured as above. Data presented as means + SEM of *n* = 2–5, analyzed by one-way ANOVA with post hoc *t*-test. Islet donors: 1, 2, 3, 5, 7 (Table S2). ns: not significant; * *p* < 0.05; ** *p* < 0.01; *** *p* < 0.001; **** *p* < 0.0001.

Since the function of EZH2 in GLT-exposed β-cells is unknown, we investigated the effect of EZH2 attenuation on GLT-induced β-cell apoptosis. For this purpose, we generated stable EZH2 knockdown (KD) INS-1E cell lines using three different shRNAs (sh), achieving ~73% mRNA knockdown with sh1 and ~56% knockdown with sh3 and sh4 (Figure 2A). These mRNA reductions led to reductions of EZH2 protein levels (Figure 2B). Next, we observed that sh3 and sh4 EZH2 KD cell lines were protected against GLT-induced apoptosis (Figure 2C). In contrast, we observed aggravated GLT-induced apoptosis using sh1 (Figure 2C). To substantiate the contrasting effects of the shRNA-induced EZH2 knockdown, we generated a heterozygous EZH2 expressing cell line (EZH2 HET) using CRISPR/Cas9 and a gRNA targeting the binding-site of the EZH2 co-factor EED required for PRC2-mediated transcriptional repression [21]. This induced a TT insertion on one EZH2-allele (Figure S3A), resulting in a premature stop-codon (Figure S3B) and a ~26% reduction in mRNA expression (Figure 2D). Similar to sh3- and sh4-expressing cell lines, EZH2 HET was protected against GLT-induced apoptosis (Figure 2E).

Interestingly, we did not observe a reduction in β-cell growth rate in EZH2 KD or HET cells (Figure S3C,D), as described in homozygous EZH2 knockout mice [30,33]. On the contrary, moderate EZH2 attenuation tended to have an accelerated growth rate (Figure S3C,D).

Finally, we investigated if two different small-molecule inhibitors of EZH2 HMT activity (EZH2i) rescued human islets from GLT-induced β-cell apoptosis. Since EZH2i induce apoptosis at high concentration in other cell types [35,36], we first tested different concentrations of the EZH2i GSK126 and GSK343 to assess inhibitor toxicity (Figure S4). We observed that 10 μmol/L EZH2i induced apoptosis in human islets (Figure S4). Next, we exposed human islets to GLT and observed induction of apoptosis as expected (Figure 2F). When exposing human islets to GLT in combination with low concentrations of either EZH2i, however, we did not observe such an increase in apoptosis (Figure 2F). Taken together, these results suggest that moderate loss of EZH2 HMT-activity protects the β-cell against GLT-induced apoptosis.

### 2.3. EZH2 Attenuation Protects the β-Cell against GLT-Induced ER Stress

**Figure 3:**
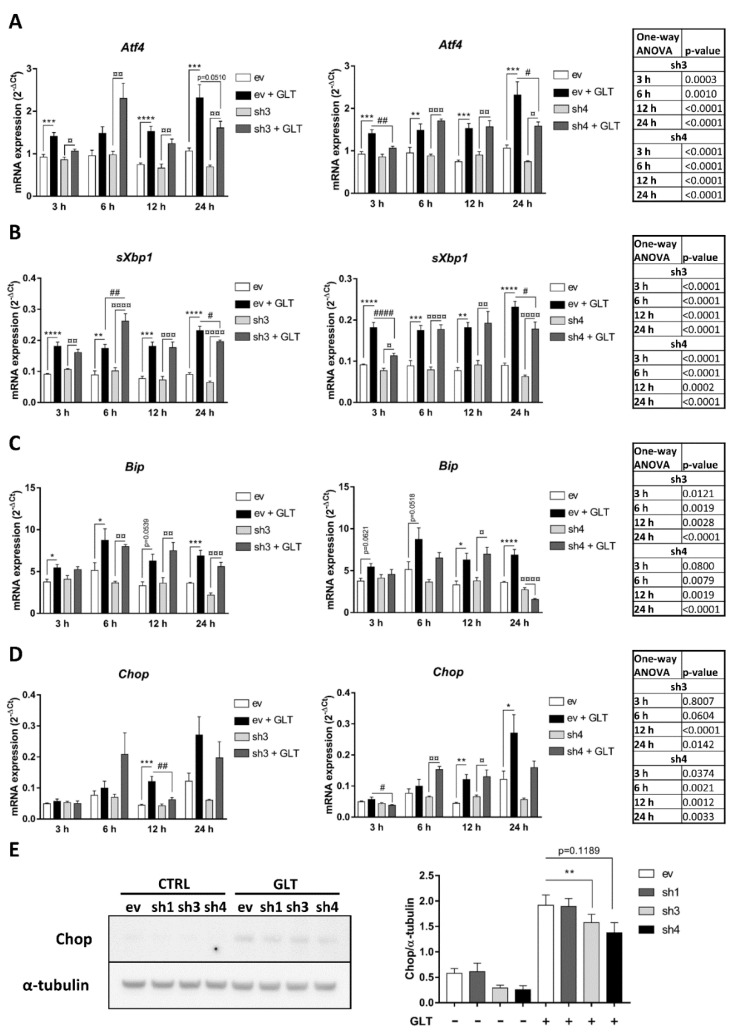
Moderate Enhancer of zeste homolog 2 (EZH2) attenuation protects against glucolipotoxicity (GLT)-induced endoplasmic reticulum (ER) stress. INS-1E cells expressing shRNAs targeting EZH2 (sh3 and -4) were exposed to 25 mmol/L glucose and 0.5 mmol/L palmitate (GLT) or vehicle for 3–24 h (**A**–**D**) and mRNA expression of ER stress markers *Activating transcription factor 4* (*Atf4*) (**A**), *Spliced X-box-binding protein-1* (*sXbp1*) (**B**), *Binding immunoglobulin protein (Bip*) (**C**) and *C/EBP homologous protein* (*Chop*) (**D**) was measured by real-time quantitative PCR. Data presented as means + SEM of *n* = 5. (**E**) INS-1E cells expressing shRNAs targeting EZH2 (sh1, -3 and -4) were exposed to 25 mmol/L glucose and 0.5 mmol/L palmitate (GLT) for 24 h and protein expression of Chop was measured by Western blot. Representative blot of *n* = 5. Data presented as means + SEM of *n* = 5. ev: INS-1E cells transfected with empty vector; sh1: INS-1E cells transfected with shRNA sequence #1; sh3: INS-1E cells transfected with shRNA sequence #3; sh4: INS-1E cells transfected with shRNA sequence #4. (**A**–**D**) analyzed by one-way ANOVA with Sidak’s multiple comparisons test. (**E**) analyzed by paired Student’s *t*-test. * *p* < 0.05, ** *p* < 0.01, *** *p* < 0.001, **** *p* < 0.0001 ev vs. ev + GLT; ^¤^ *p* < 0.05, ^¤¤^ *p* < 0.01, ^¤¤¤^ *p* < 0.001, ^¤¤¤¤^ *p* < 0.0001 sh3 vs. sh3 + GLT (**A**–**D** left panels) or sh4 vs. sh4 + GLT (**A**–**D** right panels); ^#^ *p* < 0.05, ^##^ *p* < 0.01, ^####^ *p* < 0.0001 ev + GLT vs. sh3 + GLT (**A**–**D** left panels) or ev + GLT vs. sh4 + GLT (**A**–**D** right panels).

While GLT potently induces β-cell apoptosis after 24 h exposure, mRNA expression of ER stress markers is induced by GLT as early as 3 h post exposure [16]. To assess whether the observed reduction in GLT-induced apoptosis in EZH2 KDs was associated with reduced activation of the ER stress pathway, we thus investigated different targets of the unfolded protein response at different time points by qPCR. We observed a protection against GLT-induced upregulation of *Activating transcription factor 4* (*Atf4*) and *Spliced X-box-binding protein-1* (*sXbp1*) mRNA expression in EZH2 KD cell lines sh3 and sh4 after 24 h exposure (Figure 3A,B), suggesting downregulation of Protein kinase RNA-like endoplasmic reticulum kinase (PERK) and Serine/threonine-protein kinase/endoribonuclease (IRE1) pathways, respectively. *Binding immunoglobulin protein* (*Bip*) mRNA expression, a target gene of Activating transcription factor 6 (ATF6), did not change significantly between EZH2 KDs or empty vector (ev) with the exception of sh4 after 24 h GLT-exposure, suggesting that the ATF6 pathway is not the main target of EZH2 (Figure 3C). While we did not see significant reduction of *Chop* mRNA in EZH2 KDs (Figure 3D), we did observe protection against GLT-induced upregulation of Chop protein in sh3 and sh4 (Figure 3E). Of note, Chop expression did not change significantly in sh1 compared to ev (Figure 3E), mirroring the lack of protection against GLT-induced apoptosis for this cell line (Figure 2C). Thus, we conclude that moderate EZH2 attenuation modestly protects the β-cell against GLT-induced activation of ER stress pathways.

### 2.4. EZH2 Attenuation Does Not Protect against GLT-Induced Insulin-Secretory Dysfunction

**Figure 4:**
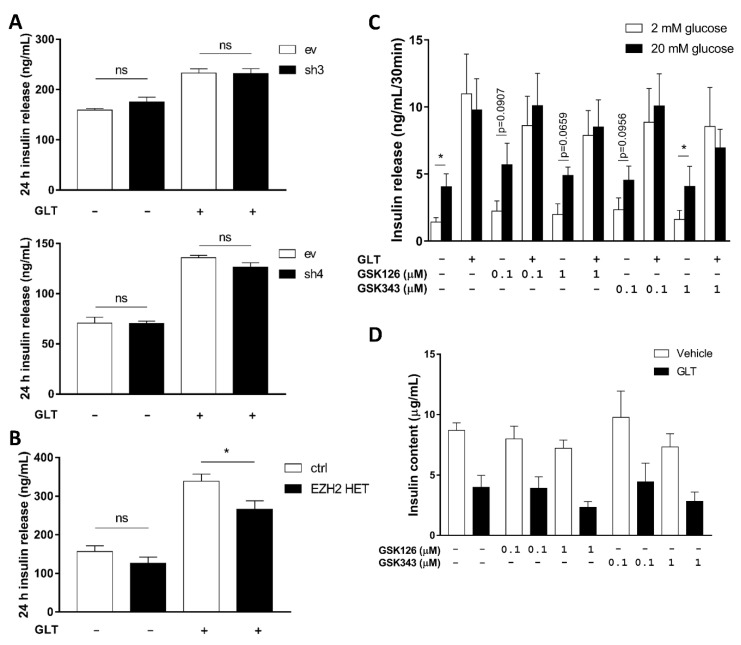
No consistent effect of Enhancer of zeste homolog 2 (EZH2) attenuation on insulin secretion and content under glucolipotoxic (GLT) conditions. Fifty-thousand INS-1E cells expressing shRNAs targeting EZH2 (sh3, upper panel and -4, lower panel; (**A**)) or gRNA targeting exon 3 of EZH2 (EZH2 HET; (**B**)) were exposed to 25 mmol/L glucose and 0.5 mmol/L palmitate (GLT) for 24 h. Insulin was detected in supernatants by ELISA. Glucose-stimulated insulin secretion (**C**) and insulin content (**D**) from twenty human islets exposed to 25 mmol/L glucose and 0.5 mmol/L palmitate (GLT) or vehicle in the presence or absence of 0.1–1 μmol/L EZH2 inhibitor GSK126 or GSK343 for 72 h. Islet donors: 6–10 (Table S2). (**A**,**B**) Data presented as means + SEM of *n* = 4, analyzed by one-way ANOVA with Sidak’s multiple comparisons test. ev: INS-1E cells transfected with empty vector; sh3: INS-1E cells transfected with shRNA sequence #3; sh4: INS-1E cells transfected with shRNA sequence #4; ctrl: failed INS-1E CRISPR clone; EZH2 HET: CRISPR/Cas9 modified INS-1E with gRNA targeting EZH2 exon 3. (**C**,**D**) Data presented as means + SEM of *n* = 5. (**C**) Analyzed by one-way ANOVA with post hoc *t*-test. ns: not significant; * *p* < 0.05.

Several studies have shown that homozygous conditional β-cell EZH2 deficiency in *RIP-Cre;Ezh2^f/f^* or *Pdx1-Cre;Ezh2^f/f^* mice leads to impaired glucose-tolerance and insulin secretion [30,31,33]. Furthermore, since GLT causes β-cell secretory dysfunction, we investigated insulin release in GLT-exposed EZH2 KD and HET cells, and in human islets treated with EZH2i. Twenty-four hour insulin release was not altered under normal conditions in EZH2 KD and -HET cells (Figure 4A,B). Only EZH2 HET cells were protected against dysregulated insulin release as assessed by 24 h accumulated insulin release in response to GLT (Figure 4B). Human islets treated with EZH2i at different concentrations secreted insulin normally in response to glucose (Figure 4C). In GLT conditions, however, EZH2i failed to normalize basal and stimulated insulin secretion (Figure 4C), and insulin content (Figure 4D). This suggests that the rescue potential of HDAC3i on insulin-secretory dysfunction likely targets other factors than EZH2.

### 2.5. EZH2 KD Prevents Downregulation of the Non-Canonical NFκB Pathway

**Figure 5:**
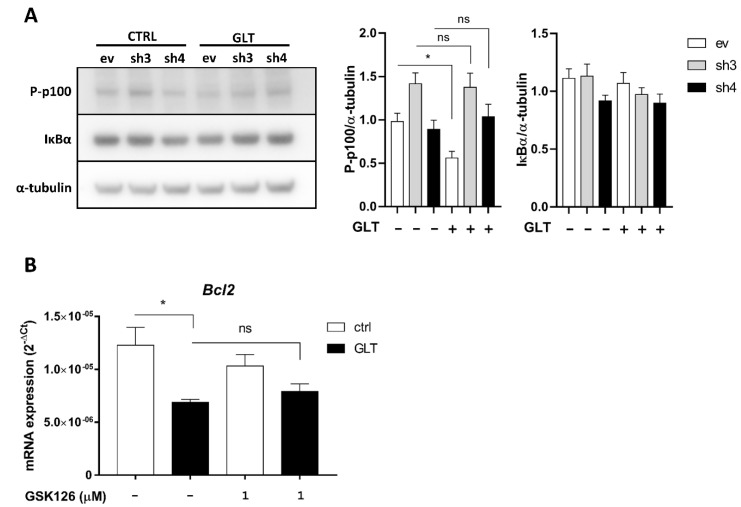
Enhancer of zeste homolog 2 (EZH2) knockdown prevents downregulation of the non-canonical Nuclear factor of kappa light polypeptide gene enhancer in B-cells (NFκB) pathway. (**A**) INS-1E cells expressing shRNAs targeting EZH2 (sh3 and -4) were exposed to 25 mmol/L glucose and 0.5 mmol/L palmitate (GLT) or vehicle (CTRL). Expression levels of phosphorylated p100 (P-p100) and nuclear factor of kappa light polypeptide gene enhancer in B-cells inhibitor, alpha (IκBα) were detected by Western blot. Data presented as means + SEM. Representative blot of *n* = 5–7, analyzed by paired Student’s *t*-test. ev: INS-1E cells transfected with empty vector; sh3: INS-1E cells transfected with shRNA sequence #3; sh4: INS-1E cells transfected with shRNA sequence #4. (**B**) One-hundred mouse islets were exposed to 25 mmol/L glucose and 0.5 mmol/L palmitate (GLT) or vehicle in the presence or absence of 1 μmol/L EZH2 inhibitor GSK126 for 48 h. *B-cell lymphoma 2* (*Bcl2*) mRNA expression was detected by real-time quantitative PCR. Data presented as means + SEM of *n* = 3, analyzed by one-way ANOVA with Sidak’s multiple comparisons test. ns: not significant; * *p* < 0.05.

Given the potential link between canonical and non-canonical NFκB activation and ER stress [27,28,29], and the role of EZH2 as a co-activator of NFκB [26], we aimed to assess the regulation of NFκB in β-cells exposed to GLT in the absence of EZH2. We used Nuclear factor of kappa light polypeptide gene enhancer in B-cells inhibitor, alpha (IκBα) and phosphorylated p100 (P-p100) as markers of activation of the canonical and non-canonical NFκB pathways, respectively, since IκBα degradation is required for p65 release and translocation to the nucleus, and since p100 requires phosphorylation in order to be processed to p52. As expected, we did not observe any changes in IκBα expression in response to GLT, nor did we observe an effect of EZH2 KD (Figure 5A). This strengthens our hypothesis that the canonical NFκB-pathway is not involved in GLT-induced β-cell apoptosis.

Interestingly, P-p100 was downregulated in ev cells exposed to GLT, suggesting a GLT-induced downregulation of the non-canonical NFκB pathway (Figure 5A). EZH2 KD abrogated GLT-mediated reduction in P-p100 (Figure 5A). Since activation of the p52 subunit can induce anti-apoptotic genes [37], we investigated mRNA expression of the anti-apoptotic p52-target *B-cell lymphoma 2* (*Bcl2*) in mouse islets exposed to GLT and/or EZH2i. *Bcl2* was downregulated in GLT-exposed islets, suggesting that a GLT-induced reduction in p52-activation leads to reduced anti-apoptotic *Bcl2*, thereby aggravating apoptosis (Figure 5B). However, we did not observe a rescue potential of *Bcl2* expression in EZH2i-treated islets (Figure 5B), suggesting that other anti-apoptotic p52-targets, such as *B-cell lymphoma-extra large* (*Bcl-xL*), contribute to protection from GLT-induced apoptosis.

## 3. Discussion

In the present study, we aimed to identify novel targets explaining HDAC3i-mediated β-cell rescue of GLT-exposed insulin-producing cells. We identified the histone/lysine N-methyltransferase EZH2 as a unique chromatin-binding regulator of genes differentially regulated by GLT and HDAC3i. Moderate, but not pronounced, EZH2 attenuation protected β-cells and islets against GLT-induced ER stress and apoptosis, likely via regulation of the non-canonical NFκB pathway. To the best of our knowledge, this is the first study describing a role for EZH2 in GLT-induced β-cell apoptosis.

EZH2 is frequently overexpressed in several cancers, promoting cancer cell-proliferation and -survival. Thus, there is a strong incentive treating cancer patients with EZH2i. Similar to our findings in the β-cell, higher concentrations of EZH2i or pronounced KD induce apoptosis [35,36] and markers of ER stress [38] in cancer models. Interestingly, evidence from cancer also suggests several EZH2–HDAC3 interaction models. EZH2 stability and HMT activity are examples of phenomena regulated by p300/CBP-associated factor (PCAF)-mediated acetylation [39], an acetyl-transferase associated with HDAC3 [40]. Additionally, EZH2 and HDAC3 form a co-repressor complex [41]. Furthermore, combined EZH2/HDAC inhibition synergistically potentiates apoptosis-induction and reduces proliferation [42]. It is worth noting that established anti-proliferative anti-cancer HDACi display anti-inflammatory properties when administered in lower concentrations [43], allowing drug repositioning. This promotes the interesting idea of possible repositioning of low-dose EZH2i, once developed in cancer, for metabolic disease.

**Figure 6:**
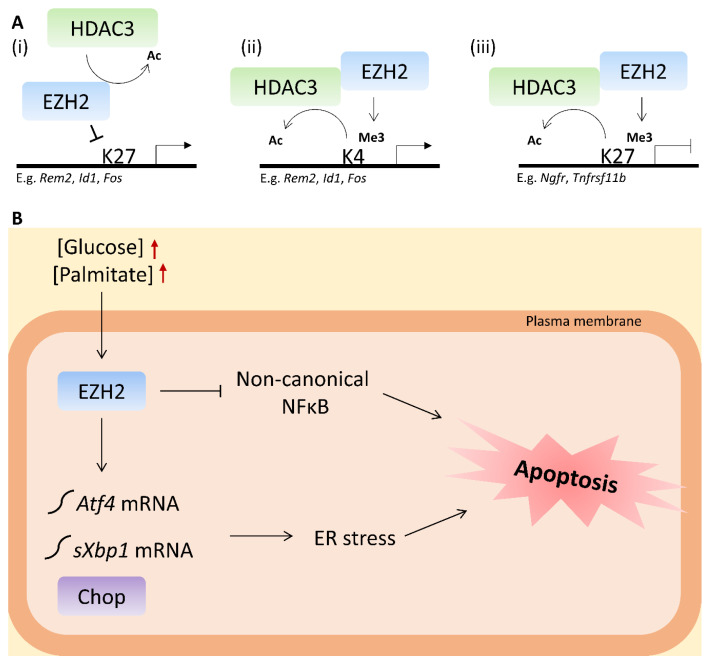
Proposed model of Enhancer of zeste homolog 2 (EZH2)/histone deacetylase 3 (HDAC3) interaction and EZH2 function in the β-cell. (**A**) Panel (i): HDAC3 targets EZH2 by removal of the activating acetyl (Ac) mark, leading to reduced EZH2 histone-lysine *N*-methyltransferase (HMT) activity on histone 3, lysine 27 (H3K27) and increased transcription of deleterious genes. Panel (ii): HDAC3 and EZH2 function as co-activators. HDAC3 catalyzes removal of the histone 3, lysine 4 (H3K4) acetyl-group, leading to EZH2 H3K4 methylation (Me3) and transcriptional activation of deleterious genes. Panel (iii): HDAC3 and EZH2 function as co-repressors. HDAC3 catalyzes the removal of the acetyl-group from H3K27, leading to EZH2 H3K27 methylation and transcriptional repression. (**B**) Proposed model of EZH2-mediated apoptosis in the β-cell. Elevated concentrations of glucose and palmitate (GLT) indicated by red arrows induce EZH2-mediated endoplasmic reticulum (ER) stress via increased *Activating transcription factor 4* (*Atf4*) and *Spliced X-box-binding protein-1* (*sXbp1*) mRNA and C/EBP homologous protein (Chop) protein expression, and EZH2 potentiates apoptotic signaling by downregulating protective non-canonical Nuclear factor of kappa light polypeptide gene enhancer in B-cells (NFκB) signaling.

The role of EZH2 in the β-cell has thus far only been investigated with respect to the EZH2 canonical repressive function as the H3K27me3-inducing catalytic subunit of PRC2 [30,31,33], and in this context as a critical regulator during pancreatic endocrine specification [30], and of β-cell dedifferentiation [31,32] and proliferation [32,33]. However, since EZH2 also displays non-canonical roles, including PCR2-independent EZH2 transcriptional activation [24,25,26], the differential EZH2 regulated gene expression pattern induced by GLT and HDAC3i suggests that EZH2 differentially regulates its targets by both transcriptional repression and activation. This notion implicates various EZH2–HDAC3 interaction models. Both EZH2 and HDAC3 target lysine 27 (K27) and lysine 4 (K4) on histone 3 (H3) in many regions to regulate transcription (Figure 6A). We observed that EZH2 target genes upregulated by GLT and potentiated by BRD3308 in general possessed functions deleterious to the β-cell, e.g., *Inhibitor of DNA Binding 1* (*Id1*) and *Musashi RNA Binding Protein 2* (*Msi2*) involved in β-cell dedifferentiation [44,45], suggesting that BRD3308 failed to counteract this GLT-induced gene expressional response. This notion is compatible with the observation that HDAC1 and −3 KD protected against cytokine-induced apoptosis, but only HDAC1 KD restored insulin release [46], and that HDAC3i failed to rescue the GLT-induced reduction in insulin stimulatory index [16]. Several genes upregulated by GLT and downregulated by BRD3308 are candidate genes that are deleterious for β-cells, e.g., *Fos Proto-Oncogene* (*Fos*), involved in formation of Activator protein 1 (AP-1) complexes [47], suggesting rescue by BRD3308. We hypothesize that HDAC3 regulates EZH2 HMT activity by EZH2 deacetylation (Figure 6(Ai)) or that EZH2 and HDAC3 function as co-activators (Figure 6(Aii)) of these genes, and that the observed protective effects of either EZH2i or HDAC3i are a result of disruption of these interactions. This, however, requires experimental validation in our cell model.

Finally, genes downregulated by GLT and upregulated by BRD3308 were generally β-cell protective candidate genes, including *Tumor Necrosis Factor Receptor Superfamily Member 11b* (*Tnfrsf11b*). *Tnfrsf11b* is involved in protection against inflammatory β-cell stress [48], and our results thus suggest that BRD3308 rescues GLT-induced downregulation of such protective candidates. We hypothesize that EZH2 and HDAC3 function as co-repressors of these genes (Figure 6(Aiii)), although this also requires experimental validation.

Since homozygous β-cell EZH2 knockout mice display reduced, while heterozygous knockouts display increased β-cell mass [30], we aimed at generating homo- and heterozygous clonal EZH2 expressing cell lines to substantiate the observed contrasts in the shRNA knockdowns. While we generated one heterozygous EZH2 expressing cell line, we were unable to generate homozygous knockdowns. Since complete deletion of EZH2 results in embryonic lethality in mice [49], we hypothesize that our inability to generate homozygous EZH2 knockout cell lines from single cells is a result of growth arrest. Taken together with the consistent phenotype observed in our three models of EZH2 deficiency (KD, HET, EZH2i) we suggest that the function of EZH2 in our models primarily ascribes to EZH2 as a PRC2-independent methyltransferase of proteins or a methyl-dependent transcription factor, since the EZH2i employed competitively inhibit the EZH2 catalytic domain by competing for the methyl-donor S-adenosyl methionine (SAM) [50,51].

We conclude from these studies that moderate inhibition of EZH2, a novel target in GLT-stressed β-cells, protects the β-cell against GLT-induced ER stress and apoptosis. The protective mechanism involves restoration of the protective non-canonical NFκB pathway (Figure 6B). We also found that EZH2 likely regulates the GLT-HDAC3-apoptosis axis in its capacity as a protein-methyltransferase or methylation-dependent transcriptional activator, but more studies are required to elucidate the primary mode of EZH2 function in metabolically stressed β-cells in vitro and in vivo to assess the translational relevance of targeting EZH2 in T2D.

## 4. Materials and Methods

### 4.1. Cell Culture and Reagents

INS-1E cells (generated and kindly provided by P. Maechler and C. Wollheim, University of Geneva, Switzerland [52,53]) were cultured in complete RPMI-1640 medium with GlutaMAX (Life Technologies, Naerum, Denmark) supplemented with 10% fetal bovine serum (FBS) (*v/v*), 100 IU/mL penicillin, 100 μg/mL streptomycin, 10 mmol/L Hepes, 50 μmol/L β-mercaptoethanol (all from Life Technologies, Naerum, Denmark) and 1 mmol/L sodium pyruvate (Sigma-Aldrich, Soeborg, Denmark) at 37 °C in a humidified atmosphere with 5% CO_2_. For GLT treatment, culture medium with 1% FBS (*v/v*) and 1% low endotoxin and fatty acid free BSA (*w/v*) (Sigma-Aldrich, Soeborg, Denmark) supplemented with 25 mmol/L glucose and 0.5 mmol/L ethanol-dissolved palmitate (both from Sigma-Aldrich, Soeborg, Denmark) conjugated to BSA at a molar ratio of 3.33:1 was used. Ethanol and BSA was used as vehicle for GLT. All experiments were performed within the passage numbers 55–80, since INS-1E display a stable phenotype between passages 40–100 [53]. All cells tested negative for Mycoplasma.

### 4.2. Small Molecule Inhibitors

HDAC3 inhibitor BRD3308 (kindly provided by E. Holson and B. K. Wagner) was dissolved in DMSO (Sigma-Aldrich, Soeborg, Denmark) and used at a concentration of 10 μmol/L. EZH2 inhibitors GSK126 (Cayman Chemical, Ann Arbour, MI, USA) and GSK343 (Sigma-Aldrich, Soeborg, Denmark) were dissolved in DMSO (Sigma-Aldrich, Soeborg, Denmark) and used at concentrations of 0.01–10 μmol/L. DMSO was used as vehicle in inhibitor experiments, and kept at a concentration ≤ 0.5%.

### 4.3. Lentiviral shRNA-Mediated EZH2 Knockdown

EZH2 knockdown in INS-1E cells was achieved using pLKO.1 Lentiviral shRNA particles (Dharmacon, Herlev, Denmark) against EZH2 mRNA (TRCN0000040073; sh1, TRCN0000039040; sh3, and TRCN0000039041; sh4), pMD2.G (12259) envelope- and psPAX2 (12260) packing-plasmids from the Tronolab (Lausanne, Switzerland) as described in [54]. pLKO.1 empty vector (Sigma-Aldrich, Soeborg, Denmark) was used as control.

### 4.4. Generation of CRISPR/Cas9-Mediated Heterozygous EZH2 Expressing Cells

INS-1E cells genome was modified using ready-to-use Lentiviral particles encoding a guide RNA (gRNA) sequence targeting EZH2 exon 3 (TargetID: RN0000261080) or a non-targeting gRNA (CRISPR12V-1EA) from Sigma-Aldrich, Soeborg, Denmark, as described in [54]. To assess successful gRNA targeting of EZH2, total RNA was isolated from single cell clones and cDNA amplified (details below) using primers binding in the introns flanking the exon of interest (Table S3). PCR product purification and Sanger Sequencing was performed by Genewiz UK (Takeley, UK). Sequences were analyzed and visualized using Unipro UGENE v1.29.0 (Unipro, Novosibirsk, Russia) [55]. Of 20 clonal cell lines, only one displayed modification in EZH2 sequence. Clones with no changes in the target sequence were thus used as controls. Translation of nucleotide to protein sequence was performed using the ExPASY Translate Tool v2 (Swiss Institute of Bioinformatics, Lausanne, Switzerland) [56].

### 4.5. Human Islets

Human islets were isolated by the European Consortium for Islet Transplantation (ECIT) Islets for Basic Research program in Milan, Italy and Geneva, Switzerland under local approval and supported by the JDRF award 31-2008-416. Islets were received fully anonymized, and originated from ten non-diabetic donors (Donor information listed in Table S2). Islets were cultured in RPMI-1640 with a final glucose concentration of 5.6 mmol/L supplemented with 10% FBS (*v/v*), 100 IU/mL penicillin and 100 μg/mL streptomycin (all from Life Technologies, Naerum, Denmark), at 37 °C in a humidified atmosphere with 5% CO_2_ for at least 2 days prior to exposure to 0.5 mmol/L palmitate and 25 mmol/L glucose in medium with 1% low endotoxin fatty acid free BSA (*w/v*) (all from Sigma-Aldrich, Soeborg, Denmark) and without FBS for 72 h.

### 4.6. Mouse Islets

Pancreata were inflated and digested with 1.4 mg/mL collagenase type 4 (Worthington Biochemical Corporation, Lakewood, CA, USA), and incubated for 30 min at 37 °C in a water bath. Islets were filtered, handpicked to purity and cultured at 37 °C in RPMI-1640 with 11.1 mM glucose supplemented with 10% FBS (*v/v*), 2 mmol/L GlutaMAX, 100 IU/mL penicillin, 100 μg/mL streptomycin and 50 μg/mL gentamycin (all from ThermoScientific, Zug, Switzerland). Islets were exposed to 0.5 mmol/L palmitate and 25 mmol/L glucose in medium with 1% low endotoxin and fatty acid free BSA (*w/v*) (all from Sigma-Aldrich, Buchs, Switzerland) and 1% FBS (*v/v*) for 48 h. All animal experiments were performed according to the Swiss veterinary law and institutional guidelines, and upon approval (accessed on 2 January 2017) by Swiss authorities (Veterinäramt, Basel-Stadt, Switzerland) within the framework of the animal experiment permit 2401.

### 4.7. mRNA Microarray

Total RNA extracted (details below) from INS-1E cells exposed to vehicle or 0.5 mmol/L palmitate, 25 mmol/L glucose (all from Sigma-Aldrich, Soeborg, Denmark) and 10 μmol/L BRD3308 (kindly provided by E. Holson and B. K. Wagner) for 6 h was spotted onto an Affymetrix GeneChip^®^ Rat Genome 230 2.0 Array (ThermoScientific, Copenhagen, Denmark). Microarray data were normalized, analyzed and visualized using R version 3.0.2 (R Foundation for Statistical Computing, Vienna, Austria), Bioconductor v2.12 [57], and the affy v1.38.1 [58], genefilter v1.42.0 [59], annotate v1.38.0 [60], gplots v2.12.1 [61], and rat2302.db v2.9.0 [62] packages. In brief, expressional data were normalized by the Robust Multi-array Average approach, filtered for ENTREZ GENE ID and UNIQUE and assigned Gene Ontology terms for each probe/Affymetrix ID. Next, the top 400 differentially expressed genes were extracted for the two models: ctrl to GLT (disease) and GLT to GLT+BRD3308 (intervention). Genes present in both models were extracted for visualization and for analysis using the ENCODE ChIP-Seq Significance Tool [34]. For visualization relative to the control condition, the control median was subtracted for each gene of interest. Microarray data were validated by real-time PCR (see below).

### 4.8. Quantitative Real-Time PCR

Total RNA was extracted using the NucleoSpin^®^ RNA kit (Macherey-Nagel, Bethlehem, PA, USA), quality and concentration measured on NanoDrop2000 (ThermoScientific, Copenhagen, Denmark), and cDNA synthesized using the iScript™ cDNA Synthesis kit (Bio-Rad, Copenhagen, Denmark). cDNA was subjected to qPCR using SYBR Green Master Mix (Applied Biosystems, Naerum, Denmark) and data acquired using the 7900HT Real-Time PCR system (Applied Biosystems, Naerum, Denmark). Primers were purchased from TAG Copenhagen (Copenhagen, Denmark). For primer sequences, see Table S4.

### 4.9. Apoptosis

Fifty thousand INS-1E cells or 25 human islets were plated in duplicates, and apoptosis was determined as accumulation of cytoplasmic histone-associated DNA-fragments using the Cell Death Detection ELISA kit (Roche, Mannheim, Germany) according to the manufacturer’s protocol.

### 4.10. Immunoblotting

Two million INS-1E cells were exposed to GLT, lysed in complete lysis buffer (50 mmol/L Tris pH8 (Sigma-Aldrich, Soeborg, Denmark), 150 mmol/L NaCl, 5 mmol/L KCl, 5 mmol/L MgCl_2_ (all from Merck, Darmstadt, Germany), 1% NP-40 (*v/v*; Sigma-Aldrich, Soeborg, Denmark), 1× Roche protease inhibitors (Roche, Mannheim, Germany) and 20 mmol/L iodoacetamide (Sigma-Aldrich, Soeborg, Denmark)), and protein concentration was measured using Bio-Rad Protein Assay Dye Reagent (Bio-Rad, Copenhagen, Denmark). Protein concentration-adjusted lysates were loaded onto SDS-PAGE gels as described in [54] and blotted with antibodies against EZH2, Chop, IκBα, phosphorylated p100 or α-tubulin (Table S5). Blots were developed using chemiluminescence. ImageJ v1.51j8 (NIH, Bethesda, MD, USA) was used for quantification.

### 4.11. Insulin Secretion

For determination of glucose-stimulated insulin secretion, 20 human islets per well in duplicate were pre-incubated in a 24-well plate in 400 μL modified Krebs-Ringer buffer (115 mmol/L NaCl, 4.7 mmol/L KCl, 1.2 mmol/L KHP_2_O_4_, 1.2 mmol/L MgSO_4_, 5 mmol/L NaHCO_3_ (all from Merck, Darmstadt, Germany), 2.6 mmol/L CaCl_2_ × 2H_2_O, 0.2% BSA (*w/v*), 2 mmol/L glutamine (all from Sigma-Aldrich, Soeborg, Denmark), 20 mmol/L Hepes (Life Technologies, Naerum, Denmark), pH adjusted to 7.4; KRBH) supplemented with 2 mmol/L glucose (Sigma-Aldrich, Soeborg, Denmark) for 1.5 h at 37 °C in a humidified atmosphere with 5% CO_2_. Islets were subsequently moved to fresh KRBH supplemented with 2 mmol/L glucose for 30 min at 37 °C, followed by collection of 200 μL supernatant. Two-hundred μL KRBH supplemented with 38 mmol/L glucose were added to wells for a final concentration of 20 mmol/L glucose for 30 min at 37 °C. Supernatants were collected and islets were lysed in complete lysis buffer. Twenty-four h insulin release was measured in supernatants from GLT or vehicle treated INS-1E or EZH2 KD and HET cells. Insulin concentrations of supernatants and lysates were determined using an in-house rat insulin ELISA [63] or Human Insulin ELISA kit (Sigma-Aldrich, Soeborg, Denmark).

### 4.12. Data Analysis

Results are presented as means + SEM. Statistical analyses were performed using GraphPad Prism 8.0 software (San Diego, CA, USA). Student’s *t*-test or one-way ANOVA with Sidak’s post hoc test were used as indicated to assess statistical significance.

### 4.13. Data and Resource Availability

Datasets generated for this study are available from the corresponding author upon reasonable request.

## Supplementary Materials

Supplementary Materials can be found at https://www.mdpi.com/1422-0067/21/21/8016/s1.

## Abbreviations

ATF	Activating transcription factor
Bcl2	B-cell lymphoma 2
Bcl-xL	B-cell lymphoma-extra large
BiP	Binding immunoglobulin protein
CHOP	C/EBP homologous protein
EED	Embryonic ectoderm development
ER	Endoplasmic reticulum
EZH2	Enhancer of zeste homolog 2
FOS	Fos Proto-Oncogene
GLT	Glucolipotoxicity
HDAC	Histone deacetylase
HMT	Histone-lysine N-methyltransferase
ID1	Inhibitor of DNA Binding 1
IκBα	Nuclear factor of kappa light polypeptide gene enhancer in B-cells inhibitor, alpha
JNK	c-Jun N-terminal kinase
IRE1	Serine/threonine-protein kinase/endoribonuclease
MSI2	Musashi RNA Binding Protein 2
NEFA	Non-esterified fatty acid
NFkB	Nuclear factor of kappa light polypeptide gene enhancer in B-cells
PERK	Protein kinase RNA-like endoplasmic reticulum kinase
PRC2	Polycomb repressive complex 2
RbAp48	Histone-Binding Protein Retinoblastoma-Binding Protein 4
SAM	S-adenosyl methionine
SUZ12	Suppressor of Zeste 12
T2D	Type 2 diabetes
TNFRSF11B	Tumor Necrosis Factor Receptor Superfamily Member 11b
sXBP1	Spliced X-box-binding protein-1
