# Supplementary material for: Enhancer of Zeste Homolog 2 (EZH2) Mediates Glucolipotoxicity-Induced Apoptosis in β-Cells
Source: Int J Mol Sci. 2020 Oct 29;21(21):8016. doi: 10.3390/ijms21218016 (PMC7672588; doi:10.3390/ijms21218016)
Supplement: Supplementary file 1 [file ijms-21-08016-s001.docx]

**SUPPLEMENTARY DATA**

**Enhancer of zeste homolog 2 (EZH2) mediates glucolipotoxicity-induced apoptosis in β-cells**

Tina Dahlby^1^, Christian Simon^2^, Marie Balslev Backe^1^, Mattias Salling Dahllöf^1^, Edward Holson^3^, Bridget K. Wagner^3^, Marianne Böni-Schnetzler^4^, Michal Tomasz Marzec^1^, Morten Lundh^1,3^, Thomas Mandrup-Pouslen^1*^

**The Supplement contains:**

**Supplementary materials and methods**

**Supplementary table 1**

**Supplementary table 2**

**Supplementary table 3**

**Supplementary table 4**

**Supplementary table 5**

**Supplementary figure 1**

**Supplementary figure 2**

**Supplementary figure 3**

**Supplementary figure 4**

**Supplementary materials and methods**

**Cell growth rate**

**For determination of cell growth rate, EZH2 KD and HET cells were manually counted after one week of culture. 3×10^6^ EZH2 HET cells were plated in T25 flasks and 6×10^6^ EZH2 KD cells were plated in in T75 flasks and cultured for seven days. Medium was replenished mid-week. After one week, cells were dissociated with Trypsin-EDTA (0.05%, ThermoScientific), collected with 3 mL complete medium and resuspended. Cell suspensions were diluted with complete medium up to 5 mL and 20 mL, respectively, and counted in a Bürker-Türk counting chamber.**

**Supplementary Tables**

**Supplementary Table 1: Fifty-two genes regulated by glucolipotoxicity (GLT) and/or the HDAC3i BRD3308 and their regulation pattern.**

| **Upregulated by GLT**  **🡪 upregulated by BRD3308** | **Upregulated by GLT**  **🡪 downregulated by BRD3308** | **Downregulated by GLT**  **🡪 downregulated by BRD3308** | **Downregulated by GLT**  **🡪 upregulated by BRD3308** |
| --- | --- | --- | --- |
| ***Dixlc1*** | ***Prkag2*** | ***Hnf4a*** | ***Stard5*** |
| ***Rem2*** | ***Nr4a2*** | ***Per2*** | ***Adora2b*** |
| ***Prok2*** | ***Etv5*** | ***Ank4*** | ***Il15*** |
| ***Id1*** | ***Hsd17b7*** | ***Serinc5*** | ***Ngfr*** |
| ***Msi2*** | ***Id2*** |  | ***Tmem100*** |
| ***Cnksr3*** | ***Mvd*** |  | ***Npepo*** |
| ***Acer1*** | ***Nr4a3*** |  | ***Rap1gap2*** |
| ***Ifi27l2b*** | ***Fst*** |  | ***Micalcl*** |
| ***Cxcl10*** | ***Fos*** |  | ***Tnfrsf11b*** |
| ***LOC360919*** |  |  | ***Cys1*** |
| ***Nupr1*** |  |  | ***Sox4*** |
| ***Sgk1*** |  |  | ***Fgfr4*** |
| ***Scd*** |  |  | ***Ndrg1*** |
| ***Hbegf*** |  |  |  |
| ***Mageh1*** |  |  |  |
| ***Vav3*** |  |  |  |
| ***Slc25a36*** |  |  |  |
| ***Rasd1*** |  |  |  |
| ***Trim72*** |  |  |  |
| ***Ppp1r17*** |  |  |  |
| ***Ppp1r3b*** |  |  |  |
| ***Cxcl11*** |  |  |  |
| ***Ros1*** |  |  |  |
| ***Areg*** |  |  |  |
| ***Fut1*** |  |  |  |
| ***Inhba*** |  |  |  |

**Supplementary Table 2: Human islet donor information.**

| **Islet isolation center** | Milan | Milan | Milan | Milan | Geneva | Geneva | Milan | Geneva | Milan | Milan |  |
| --- | --- | --- | --- | --- | --- | --- | --- | --- | --- | --- | --- |
| **Source** | ECIT | ECIT | ECIT | ECIT | ECIT | ECIT | ECIT | ECIT | ECIT | ECIT |  |
| **Viability (%)** | 95 | 95 | 95 | 90 | 90 | n/a | 90 | 90 | 95 | 95 | **92.8 ± 2.6** |
| **Purity**  **(%)** | 90 | 80 | 70 | 85 | 80 | 94 | 80 | 84 | 80 | 80 | **82.3 ± 6.5** |
| **History of diabetes?** | No | No | No | No | No | No | No | No | No | No |  |
| **Random blood glucose (mg/dL)** | 136 | 148 | 130 | 106 | 138 | 68.4 | 228 | 102 | 216 | 363 | **163.5 ± 85.4** |
| **BMI (kg/m^2^)** | 23.6 | 23.1 | 24.8 | 25.1 | 30.0 | 21.5 | 29.4 | 23.7 | 22.7 | 29.0 | **25.3 ± 3.1** |
| **Sex** | F | M | M | M | M | F | F | F | M | F | **5F/5M** |
| **Age (y)** | 60 | 34 | 50 | 52 | 59 | 53 | 56 | 59 | 60 | 64 | **54.7 ± 8.4** |
| **Identifier** | HP1244 | HP1259 | HP1260 | HP1261 | HI-6 | HI-8 | HP1286 | HI-9 | HP1291 | HP1301 |  |
| **Donor no.** | **1** | **2** | **3** | **4** | **5** | **6** | **7** | **8** | **9** | **10** | **Mean ± SD** |

**Supplementary Table 3: Primers used for EZH2 sequencing PCR reaction and Sanger sequencing.** Primer sets (1) and (2) were designed to bind the introns flanking exon 3 of EZH2.

| Target | Forward (5’-…-3’) | Reverse (5’-…-3’) | Target exon | Intron | Product size (bp) |
| --- | --- | --- | --- | --- | --- |
| *EZH2 (1)* | AGTATACCTTCGCACTGCTTGT | GCTGCCATTGATATACACTGAAAC | 3 | 2&3 | 603 |
| *EZH2 (2)* | TCAGTATACCTTCGCACTGCT | AGGCTGCCATTGATATACACTGAA | 3 | 2&3 | 607 |

**Supplementary Table 4: Rat specific primers used for gene expression analysis.**

| Target | Forward (5’-…-3’) | Reverse (5’-…-3’) |
| --- | --- | --- |
| *Chop* | CAGCGACAGAGCCAAAATAAC | TGTGGTGGTGTATGAAGATGC |
| *Trim72* | AGTGTAGCAGTCCTGGAGCAT | GCTTCACGGTCCAAAGAACT |
| *Hbegf* | ATCCACGGAGAGTGCAGATAC | CCAAGACGGTAGTGTGGTCAT |
| *Adora2b* | GACTTTCACAGCTGCCTCTTC | GTGTTCCAGTGACCAAACCTT |
| *Il15* | AAGAAACGTGCTCTACCTTGC | CGTGAAGTTTCTCTCCTCCAG |
| *Fst* | CCTCAAGGCCAGATGTAAAGA | GGCATTATTGGTCTGATCCAC |
| *Fos* | GAGTGGTGAAGACCATGTCAG | GGATTCTCCGTTTCTCTTCCT |
| *Ezh2* | GGAGACGATCCTGATGAAAGA | GCCCTTATCTGGAAACATTGA |
| *Bip* | CTGGCACTATTGCTGGACTG | CCACCACTTCAAAGACACCA |
| *Atf4* | GTTGGTCAGTGCCTCAGACA | CATTCGAAACAGAGCATCGA |
| *sXbp1* | CTGAGTCCGAATCAGGTGCAG | ATCCATGGGAAGATGTTCTGG |
| *HPRT1* | GCAGACTTTGCTTTCCTT | CCGCTGTCTTTTAGGCTT |

**Supplementary Table 5: Antibodies used for immunoblotting.**

| **Antigen** | **Manufacturer** | **Catalog number** | **Molecular weight** |
| --- | --- | --- | --- |
| EZH2 | BD Biosciences | 612666 | 91 kDa |
| CHOP | Cell Signaling | 5554 | 27 kDa |
| IкBα | Santa Cruz Biotechnologies | sc-371 | 37 kDa |
| P-p100 | Sigma | SAB4301419 | 100 kDa |
| α-tubulin | Sigma | T6074 | 50 kDa |
| β-actin | Abcam | ab6276 | 42 kDa |
| HRP-anti-mouse | Cell Signaling | 7076 |  |
| HRP-anti-rabbit | Cell Signaling | 7074 |  |

**Supplementary Figures**

**
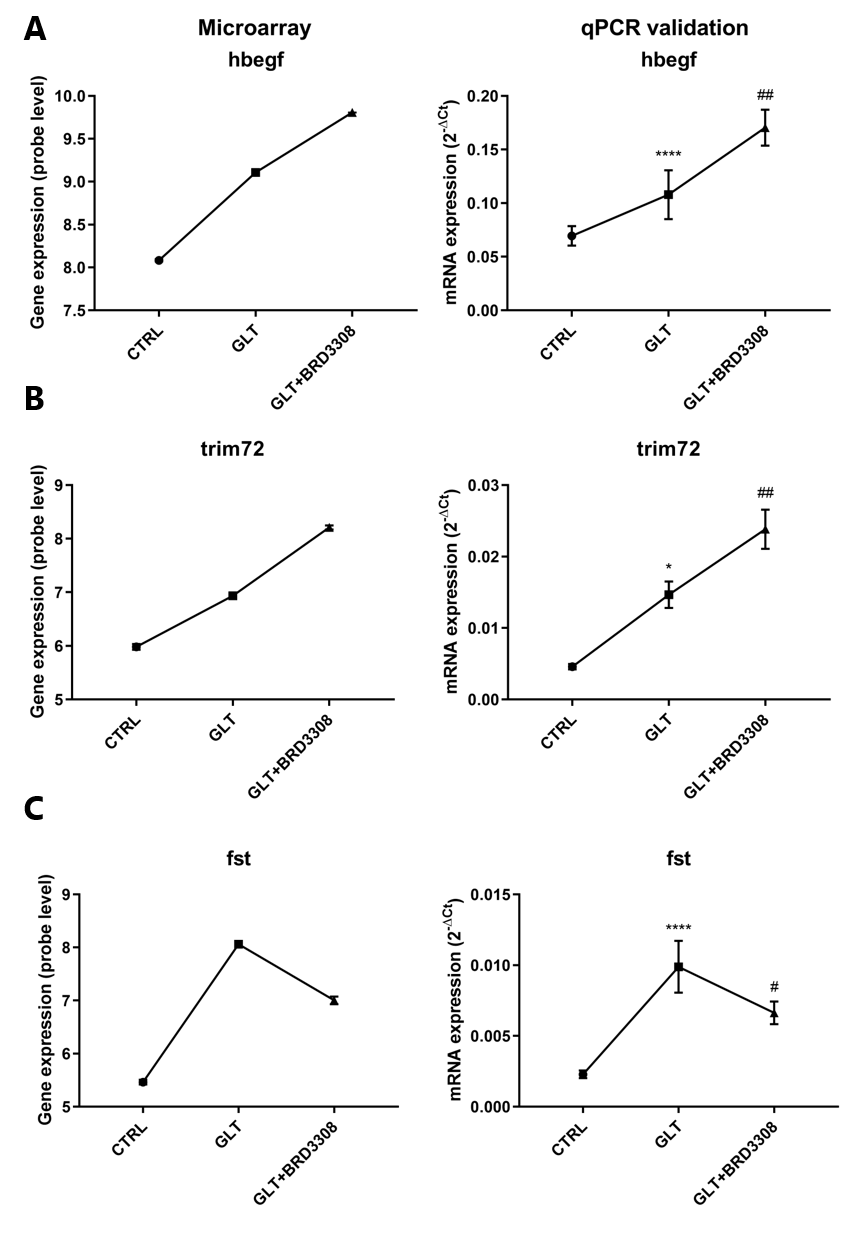
**

**
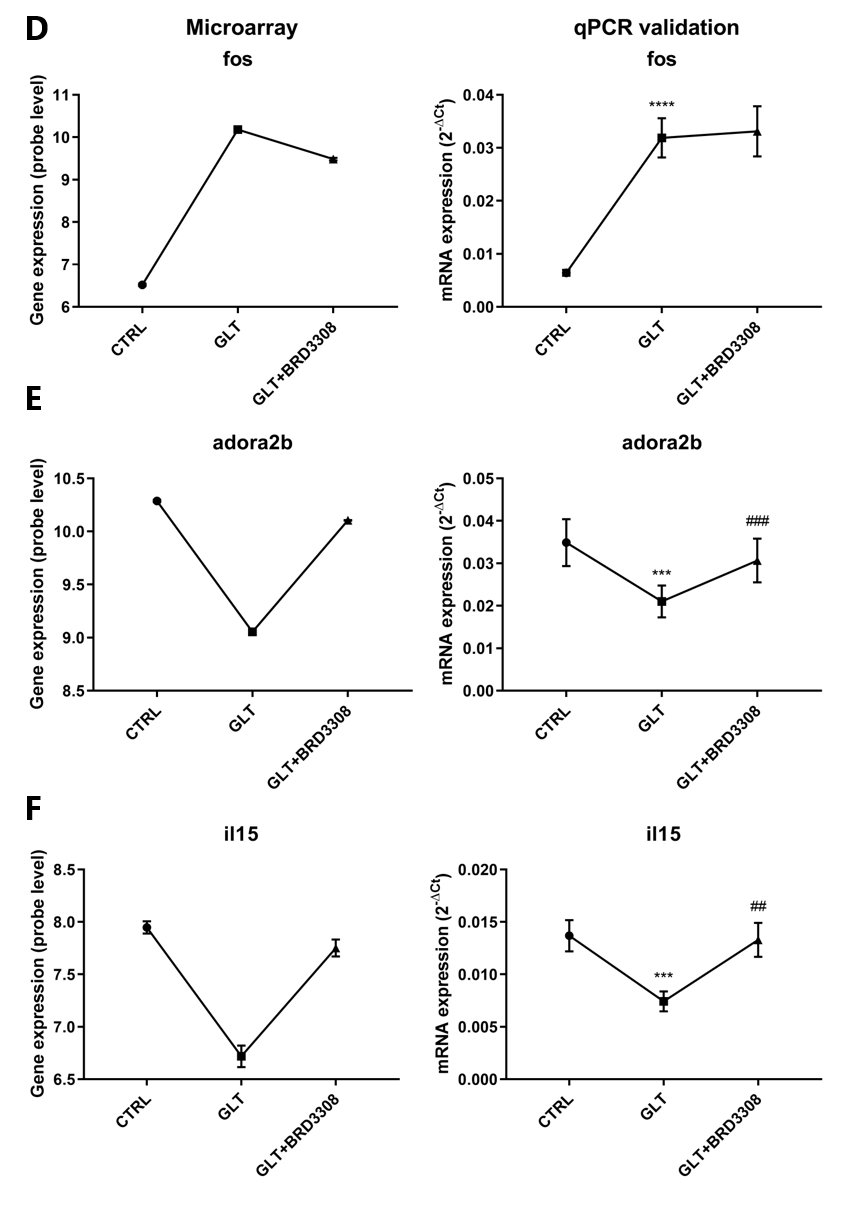
**

**Supplementary Figure 1: Validation of mRNA microarray expression patterns by qPCR. A.-F.** mRNA expression from INS-1E cells exposed to 25 mM glucose and 0.5 mM palmitate (GLT) with or without 10 μM HDAC3 inhibitor BRD3308 of selected candidate genes from microarray analysis (left panels) was analyzed by qPCR for microarray validation (right panels). Data presented as mean ± SEM of triplicates of n=1 (microarray) or n=6 (qPCR) and analyzed by paired Student’s *t*-test. * p<0.05, *** p<0.001, **** p<0.0001 vs CTRL; # p<0.05, ## p<0.01, ### p<0.001 vs GLT.

**
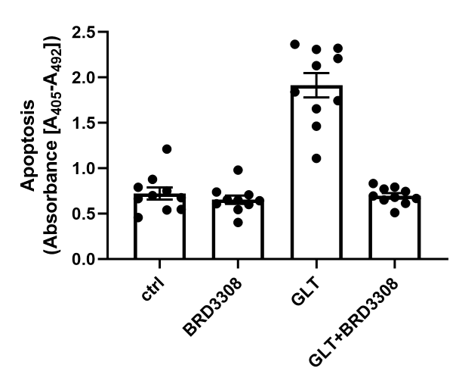
**

**Supplementary Figure 2: GLT-induced apoptosis in the presence or absence of 10 uM HDAC3i BRD3308.** To confirm GLT and BRD3308 regulation of apoptosis in our model system, 50.000 INS-1E cells were exposed to 25 mM glucose and 0.5 mM palmitate for 24 h and apoptosis was detected as cytoplasmic accumulation of mono- and oligonucleosomes. Data presented as means ± SEM of n=10.

**
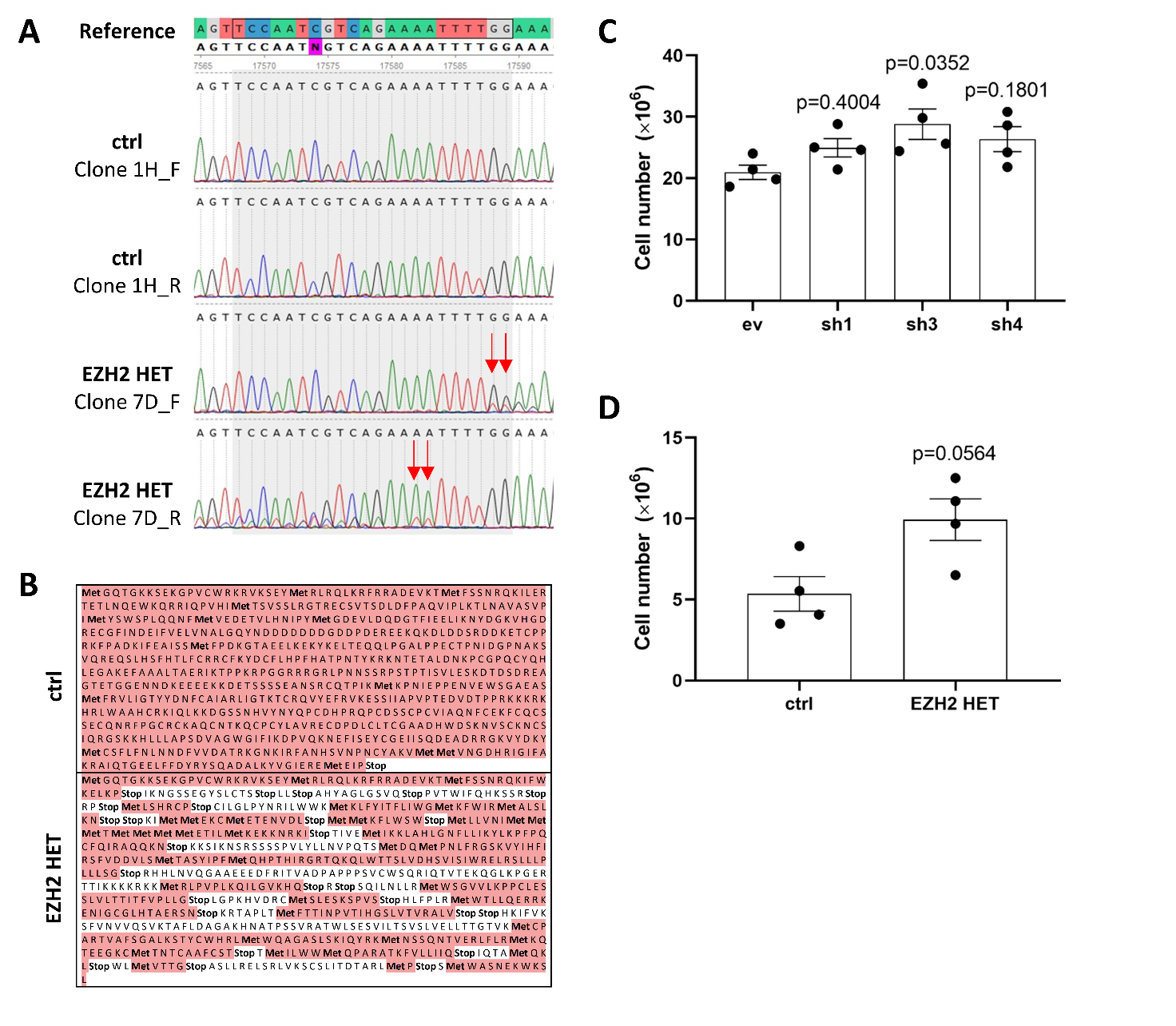
**

**Supplementary Figure 3: Characteristics of EZH2 depleted cells. A.** Sequencing of CRISPR clones. Ctrl: Failed clone. EZH2 HET: Clone with TT insertion (red arrows). F indicates sequencing with forward primer, R indicates sequencing with reverse primer. **B.** Translation of sequence from A. EZH2 HET induces a premature stop codon. **C.** EZH2 KD cell number after one week culture (T75, 6 million cells plated). Data presented as means ± SEM of n=4, analyzed by one-way ANOVA with Sidak’s multiple comparisons test. **D.** EZH2 HET cell number after one week culture (T25, 3 million cells plated). Data presented as mean ± SEM of n=4, analyzed by paired Student’s *t*-test.

**
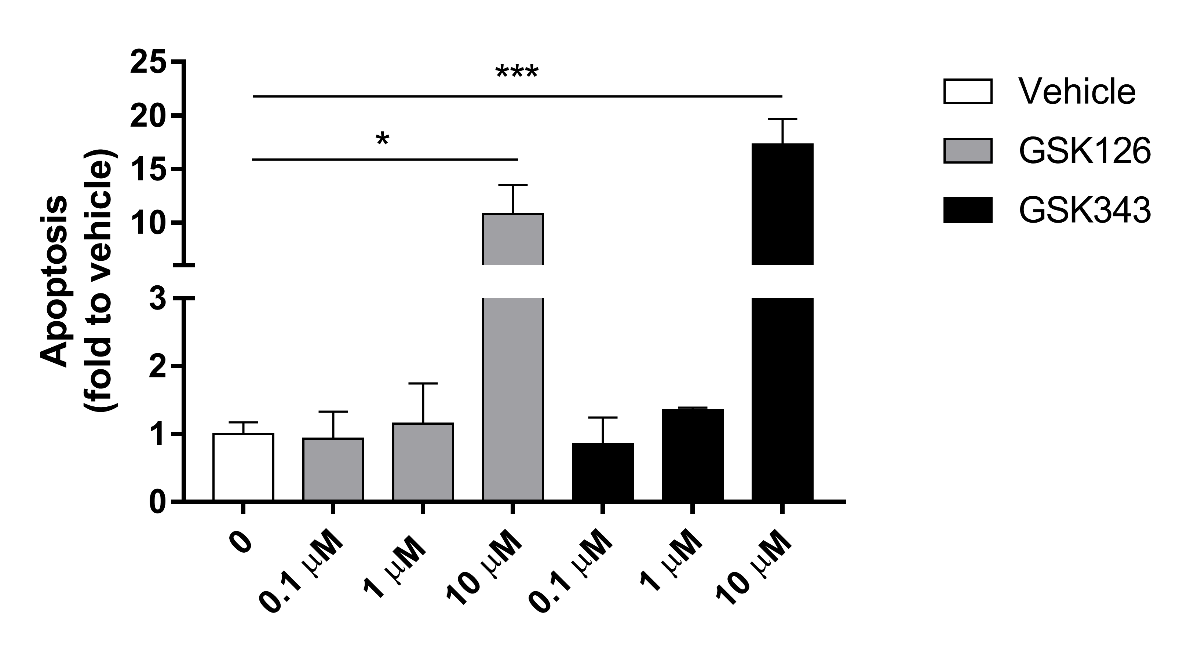
**

**Supplementary figure 4: EZH2i-induced apoptosis in human islets.** Twenty-five human islets in duplicate were exposed to 0.1-10 μM EZH2 inhibitor GSK126 or GSK343 for 72 h. Apoptosis was detected as cytoplasmic accumulation of mono- and oligonucleosomes. Data presented as means + SEM of n=3, analyzed by one-way ANOVA with Dunnett’s multiple comparisons test. Islet donors: 2, 3 and 4 (Supplementary table 2). * p<0.05; *** p<0.001.
